# Supplementary material for: Leading causes of death in Asian Indians in the United States (2005–2017)
Source: PLoS One. 2022 Aug 10;17(8):e0271375. doi: 10.1371/journal.pone.0271375 (PMC9365163; doi:10.1371/journal.pone.0271375)
Supplement: S1 Table — (DOCX) [file pone.0271375.s001.docx]

**Supplemental Table 1. Annual mortality ratio for leading causes of death in Asian Indians and non-Hispanic Whites in the United States by nativity, 2005-2017**

| Nativity | | | | | | | | | | | | | | | | | | | | |
| --- | --- | --- | --- | --- | --- | --- | --- | --- | --- | --- | --- | --- | --- | --- | --- | --- | --- | --- | --- | --- |
|  | Heart Diseases | | | | Malignant Neoplasms | | | | Cerebrovascular Disease | | | | Diabetes Mellitus | | | | Influenza & Pneumonia | | | |
|  | AI Overall | AI US-born | AI Foreign-born | NHW | AI Overall | AI US-born | AI Foreign-born | NHW | AI Overall | AI US-born | AI Foreign-born | NHW | AI Overall | AI US-born | AI Foreign-born | NHW | AI Overall | AI US-born | AI Foreign-born | NHW |
| 2005 | 95.3 (89.5-101.5) | 60.5 (36.4-96.9) | 96.9 (90.9-104.2) | 218.2 (217.6-218.8) | 54.4 (50-59.1) | 43.5 (24.2-75.1) | 54.9 (50.4-60.7) | 200.6 (200-201.2) | 16.7 (14.3-19.4) | 7.5 (1.4-27.7) | 17.2 (14.7-21.2) | 48.2 (47.9-48.5) | 16.5 (14.1-19.2) | 15.8 (5.7-39) | 16.5 (14-20.5) | 32.8 (32.6-33) | 13.3 (11.1-15.8) | 10.7 (3.3-31.3) | 13.4 (11.2-17.2) | 38.1 (37.8-38.3) |
| 2006 | 85.2 (79.8-90.8) | 87.1 (51.5-140.2) | 87.8 (82.2-95) | 188.3 (187.8-188.9) | 58.2 (53.8-63) | 66 (35.7-114.1) | 59.3 (54.7-65.6) | 188 (187.5-188.6) | 20.4 (17.8-23.3) | 13.4 (3-42.8) | 21 (18.3-25.7) | 41 (40.7-41.2) | 17.5 (15.1-20.2) | 17.3 (5.3-47.5) | 17.6 (15.1-22.1) | 29 (28.8-29.2) | 15.2 (12.9-17.7) | 2.1 (0.4-24.9) | 16.9 (13.9-21.9) | 32 (31.8-32.3) |
| 2007 | 83.3 (78.4-88.5) | 42.2 (22.4-78.2) | 85 (79.9-91.5) | 181.2 (180.6-181.7) | 54 (50.1-58.3) | 35.7 (17.6-70.3) | 54.9 (50.8-60.5) | 185.4 (184.9-186) | 13.3 (11.4-15.5) | 18.2 (4-53.3) | 13.5 (11.6-17.4) | 40 (39.7-40.2) | 16.8 (14.5-19.2) | 10 (1.6-38.4) | 16.9 (14.6-20.9) | 28.5 (28.3-28.7) | 11.7 (9.9-13.8) | 3 (0.3-26.7) | 11.9 (10.1-15.7) | 30.7 (30.5-30.9) |
| 2008 | 71.6 (67.3-76.1) | 25.6 (12.6-48.9) | 73.5 (68.8-79.4) | 176 (175.5-176.5) | 51.5 (47.8-55.4) | 46.7 (26.4-77.8) | 51.9 (48.1-56.9) | 180.7 (180.2-181.2) | 16.9 (14.8-19.2) | 1.7 (0.2-15.6) | 17.2 (15.1-20.8) | 38.4 (38.2-38.6) | 16.5 (14.5-18.9) | 22.4 (8.8-47.8) | 16.4 (14.3-19.9) | 27.9 (27.7-28.1) | 12.9 (11.1-14.9) | 10.7 (2.7-30.5) | 12.5 (10.7-15.8) | 31.2 (30.9-31.4) |
| 2009 | 75.4 (71.1-79.9) | 57.9 (32.4-98.6) | 76.2 (71.8-81.8) | 167.3 (166.8-167.8) | 54.2 (50.6-58.1) | 42.6 (21.4-79.3) | 54.7 (51-59.7) | 177.6 (177.1-178.2) | 14.9 (13-17) | 10.9 (13.0-17.0) | 14.9 (13-18.4) | 36.5 (36.3-36.7) | 14.8 (12.9-16.9) | 15 (5.6-39.9) | 14.5 (12.6-18) | 27.1 (26.9-27.3) | 16.2 (14.3-18.4) | 3.6 (0.7-24.3) | 16.8 (14.7-20.4) | 29.9 (29.7-30.1) |
| 2010 | 73.4 (69.3-77.7) | 61.9 (38.3-97.1) | 73.6 (69.5-78.8) | 164.1 (163.6-164.6) | 57.2 (53.5-61) | 41.6 (23.4-71.2) | 57.4 (53.6-62.2) | 175.4 (174.9-175.9) | 16.4 (14.6-18.5) | 25 (10.9-51.5) | 16.1 (14.2-19.3) | 36.1 (35.9-36.4) | 15.2 (13.4-17.3) | 11.3 (2.4-34.4) | 15.1 (13.3-18.3) | 26.7 (26.5-26.9) | 13.4 (11.7-15.3) | 16.4 (5.5-40.3) | 13.3 (11.5-16.4) | 29 (28.8-29.2) |
| 2011 | 69.1 (65.3-73.1) | 27.3 (15.6-45.6) | 71 (67-77) | 161 (160.5-161.4) | 57.6 (54-61.3) | 43.1 (26.6-66.7) | 58.2 (54.5-63.9) | 171.9 (171.4-172.4) | 16 (14.2-18) | 2.2 (0.2-12.2) | 16.6 (14.7-21) | 35.3 (35.1-35.5) | 16.2 (14.4-18.2) | 2.6 (0.5-12.4) | 16.7 (14.7-21.1) | 27.9 (27.7-28.1) | 14.3 (12.6-16.2) | 0.6 (0.3-9.7) | 14.6 (12.9-19) | 30.5 (30.3-30.7) |
| 2012 | 69.4 (65.8-73.2) | 18.1 (9.2-33.8) | 71 (67.3-76.8) | 152 (151.6-152.5) | 54.3 (51-57.7) | 29.9 (16.8-50.3) | 55.1 (51.8-60.6) | 160.6 (160.2-161.1) | 15.1 (13.4-17) | 8.8 (3-22.3) | 15.3 (13.6-19.6) | 33.3 (33.1-33.5) | 17.5 (15.7-19.5) | 4.7 (0.8-17) | 17.9 (16-22.3) | 26.2 (26-26.4) | 14.4 (12.8-16.2) | 9.5 (3-23.9) | 14.3 (12.7-18.6) | 27.9 (27.7-28.1) |
| 2013 | 74.3 (70.7-78) | 22.2 (12.1-38.9) | 76.1 (72.4-80.4) | 150.7 (150.3-151.2) | 56.4 (53.2-59.7) | 33.8 (20.7-53.3) | 57.2 (53.9-61.1) | 157.6 (157.2-158.1) | 15.2 (13.6-16.9) | 3.3 (1-12.7) | 15.5 (13.9-17.7) | 32.7 (32.5-32.9) | 18.2 (16.4-20.1) | 5.3 (1.2-16.7) | 18.6 (16.7-21) | 26.5 (26.3-26.7) | 16.7 (15-18.5) | 1.3 (0.2-10.3) | 17.2 (15.4-19.6) | 29.9 (29.7-30.1) |
| 2014 | 70.5 (67.1-74) | 46.2 (29.8-69.8) | 71.1 (67.7-75.2) | 147.5 (147-147.9) | 55.1 (52.1-58.2) | 36.7 (21.6-59.5) | 55.7 (52.6-59.4) | 155.8 (155.3-156.2) | 15 (13.5-16.7) | 5.6 (1.7-17.4) | 15.3 (13.7-17.6) | 33.2 (33-33.4) | 15.6 (14.1-17.4) | 1.2 (0.1-11.7) | 15.8 (14.2-18.2) | 27 (26.8-27.2) | 16.5 (14.9-18.3) | 13.3 (4.9-30.2) | 16.4 (14.8-18.8) | 29.2 (29-29.4) |
| 2015 | 74.7 (71.3-78.2) | 37.1 (22.9-58.1) | 75.7 (72.2-79.5) | 147.8 (147.3-148.2) | 67.4 (64.1-70.8) | 50.9 (33.4-75.4) | 67.9 (64.5-71.6) | 153.4 (152.9-153.8) | 17.3 (15.8-19.1) | 6 (2-17.2) | 17.7 (16-19.7) | 34.2 (34-34.4) | 18.4 (16.8-20.2) | 17 (7.4-34) | 18.4 (16.7-20.5) | 27.8 (27.6-28) | 18.2 (16.6-20) | 6.4 (1.7-18.8) | 18.3 (16.6-20.4) | 30 (29.8-30.2) |
| 2016 | 77.2 (73.8-80.6) | 33.6 (20.9-52.5) | 77.9 (74.5-81.7) | 145.8 (145.3-146.2) | 65.4 (62.3-68.7) | 31.2 (18.1-51) | 66.3 (63.1-69.9) | 152.9 (152.4-153.3) | 19.3 (17.6-21) | 3.1 (0.5-13.3) | 19.5 (17.9-21.6) | 34.4 (34.2-34.6) | 19.5 (17.8-21.3) | 13.6 (5.1-29.5) | 19.6 (17.9-21.7) | 28.2 (28-28.4) | 18.2 (16.6-20) | 5.8 (1.9-16.6) | 18.7 (17-20.8) | 28.5 (28.3-28.7) |
| 2017 | 76.8 (73.5-80.2) | 29.7 (18.7-46) | 78.4 (75-82.4) | 142.9 (142.5-143.3) | 63.7 (60.7-66.9) | 38.3 (25.2-56.6) | 64.6 (61.5-68.5) | 147.4 (146.9-147.8) | 18 (16.5-19.7) | 8.2 (3.4-18.6) | 18.4 (16.9-20.9) | 34.3 (34.1-34.5) | 21.2 (19.5-23.1) | 9.9 (4.3-21) | 21.6 (19.8-24.2) | 28.7 (28.5-28.9) | 19.3 (17.6-21) | 5.8 (1.9-15.6) | 19.6 (17.9-22.1) | 29.4 (29.2-29.6) |
|  | Heart Diseases | | | | Malignant Neoplasms | | | | Cerebrovascular Disease | | | | Diabetes Mellitus | | | | Influenza & Pneumonia | | | |
|  | AI Overall | AI US-born | AI Foreign-born | NHW | AI Overall | AI US-born | AI Foreign-born | NHW | AI Overall | AI US-born | AI Foreign-born | NHW | AI Overall | AI US-born | AI Foreign-born | NHW | AI Overall | AI US-born | AI Foreign-born | NHW |
| 2005 | 8.3 (6.7-10.3) | 12.8 (4-34.9) | 7.9 (6.2-11.3) | 44 (43.7-44.2) | 18.6 (16.5-21) | 24.6 (13.6-46.9) | 19.9 (17.3-24) | 57.2 (56.9-57.5) | 10 (8.2-12.1) | 7.6 (1.6-27.5) | 10 (8.2-13.5) | 30 (29.8-30.2) | 6 (4.6-7.7) | 1.6 (0.1-18.8) | 6.1 (4.7-9.4) | 50.4 (50.1-50.7) | 6.6 (5.1-8.3) | 3.1 (0.5-20.5) | 6.6 (5.2-9.9) | 20.2 (20.1-20.4) |
| 2006 | 7.4 (5.9-9.2) | 1.3 (0.3-24.1) | 7.5 (5.9-11.4) | 39.9 (39.7-40.2) | 19.3 (17.1-21.8) | 22.8 (10.1-51.9) | 20.2 (17-25.3) | 56.5 (56.2-56.9) | 11.7 (9.8-13.9) | 8.1 (1.8-33.1) | 11.9 (9.9-16) | 28 (27.8-28.2) | 7.8 (6.3-9.7) | 11.1 (1.8-40.4) | 7.9 (6.3-11.8) | 44.1 (43.8-44.4) | 8.7 (7-10.7) | 6.2 (1.2-30.4) | 9.7 (7.1-14.4) | 18.3 (18.1-18.4) |
| 2007 | 7.3 (5.9-8.9) | 6.7 (0.9-32.7) | 8.3 (6.1-12.6) | 40.6 (40.3-40.8) | 19.3 (17.2-21.6) | 18.4 (10.6-42.5) | 19.1 (16.8-23.3) | 57.7 (57.4-58) | 9.7 (8.2-11.6) | 3.4 (0.3-27.4) | 9.8 (8.2-13.5) | 28 (27.8-28.2) | 4.7 (3.5-6) | 2.2 (0.1-26.1) | 4.7 (3.6-8.1) | 44.7 (44.4-44.9) | 6.8 (5.4-8.4) | 9.4 (1.9-36.1) | 6.8 (5.4-10.4) | 18.4 (18.2-18.6) |
| 2008 | 7.3 (6-8.8) | 7 (1.4-24.1) | 7.3 (5.9-10.3) | 42.4 (42.2-42.7) | 18.6 (16.7-20.8) | 12.2 (5.6-28.5) | 18.1 (15.9-21.6) | 57 (56.6-57.3) | 9.5 (8-11.2) | 3.1 (0.4-17.6) | 9.4 (7.9-12.6) | 27.8 (27.6-28.1) | 6.1 (4.9-7.5) | 6 (0.6-23.9) | 6.1 (4.9-9) | 47.9 (47.7-48.2) | 5.9 (4.7-7.4) | 0 (0-14) | 6.1 (4.8-9) | 17.4 (17.2-17.5) |
| 2009 | 7.9 (6.6-9.4) | 7.2 (47150) | 7.6 (6.2-10.6) | 41 (40.8-41.3) | 17.9 (16-19.9) | 18.1 (9.3-41.1) | 18.4 (15.8-22.4) | 55 (54.7-55.3) | 9.8 (8.3-11.4) | 3.7 (0.8-24.4) | 9.7 (8.2-12.8) | 26.9 (26.7-27.1) | 6 (4.8-7.4) | 7.3 (0.9-31.9) | 6 (4.8-8.9) | 45.9 (45.7-46.2) | 6.7 (5.4-8.1) | 0.1 (0-21) | 6.8 (5.5-9.8) | 17.2 (17-17.3) |
| 2010 | 8.5 (7.2-10) | 11.7 (2.6-34.7) | 8.2 (6.9-11) | 42.7 (42.5-43) | 18.4 (16.6-20.4) | 12.7 (7.6-29) | 19 (16.8-22.4) | 56.4 (56.1-56.7) | 10.8 (9.3-12.6) | 13.5 (3.7-36.8) | 10.7 (9.1-13.6) | 27.1 (26.9-27.3) | 5.3 (4.2-6.6) | 12.1 (2.8-35.2) | 5.1 (4-7.7) | 45.3 (45.1-45.6) | 6.7 (5.5-8.1) | 21.7 (8.4-47.9) | 6.3 (5.1-9) | 17.6 (17.5-17.8) |
| 2011 | 9.7 (8.3-11.3) | 1.6 (0.4-10.7) | 9.9 (8.4-14.1) | 43.1 (42.8-43.3) | 17.7 (16-19.6) | 14.7 (8.6-26.6) | 18.2 (16.2-22.7) | 58 (57.7-58.3) | 9.4 (8.0-11.0) | 1.9 (0.2-11.8) | 9.6 (8.1-13.8) | 27.4 (27.2-27.6) | 6.7 (5.6-8) | 1.4 (0.2-10.7) | 6.9 (5.7-11) | 46 (45.7-46.2) | 6.4 (5.3-7.8) | 0.9 (0.1-10.1) | 6.6 (5.4-10.7) | 16.4 (16.2-16.5) |
| 2012 | 9 (7.8-10.5) | 5.6 (1.5-17.2) | 8.8 (7.6-12.9) | 41.2 (41-41.5) | 19 (17.2-20.8) | 18.3 (10-33.1) | 21.8 (18.6-27.1) | 57.3 (57-57.6) | 8.7 (7.4-10.1) | 2.2 (0.6-11.9) | 8.8 (7.5-12.9) | 26.3 (26.1-26.5) | 5.1 (4.2-6.2) | 4.1 (0.6-15.7) | 5.1 (4.1-9.1) | 42.7 (42.5-43) | 5.7 (4.7-6.9) | 0 (0-9.7) | 5.9 (4.9-9.9) | 15.4 (15.3-15.6) |
| 2013 | 11 (9.7-12.5) | 3.1 (0.2-14.1) | 11.7 (10.1-13.9) | 42.1 (41.8-42.3) | 17.4 (15.8-19.2) | 18.7 (11.2-31.9) | 17.4 (15.5-19.9) | 57.6 (57.3-57.9) | 9 (7.8-10.3) | 6.8 (2.1-18.4) | 9 (7.8-10.9) | 26.7 (26.5-26.9) | 6.4 (5.4-7.5) | 2.8 (0.6-12.5) | 6.4 (5.4-8.1) | 43.4 (43.2-43.7) | 6.6 (5.5-7.8) | 6.3 (1.2-19.4) | 6.6 (5.5-8.4) | 15.7 (15.6-15.9) |
| 2014 | 11.9 (10.6-13.4) | 10.6 (4.4-24.2) | 12 (10.6-14.2) | 44.9 (44.7-45.2) | 18.4 (16.8-20.1) | 20.3 (11.7-35.7) | 18.5 (16.6-21) | 59.1 (58.8-59.4) | 9.2 (8-10.5) | 9.6 (2.9-24.9) | 9.1 (7.9-11.1) | 26.9 (26.7-27.1) | 5.9 (4.9-6.9) | 5.9 (0.8-20.7) | 5.8 (4.9-7.6) | 42 (41.8-42.2) | 7.3 (6.2-8.5) | 3.8 (0.3-16.8) | 7.4 (6.3-9.3) | 15.9 (15.8-16.1) |
| 2015 | 14.8 (13.3-16.4) | 12.4 (5.3-26.5) | 14.9 (13.3-16.8) | 50.7 (50.4-50.9) | 21.9 (20.2-23.7) | 14.8 (9-26.4) | 22.9 (20.7-25.4) | 62.3 (62-62.7) | 11 (9.8-12.4) | 16 (7.3-31.8) | 10.7 (9.5-12.4) | 27.5 (27.3-27.7) | 5.6 (4.8-6.6) | 6.2 (1.5-18.6) | 5.6 (4.7-7) | 43.4 (43.2-43.7) | 7.9 (6.8-9.1) | 7.9 (2.4-21) | 7.9 (6.8-9.4) | 16.3 (16.2-16.5) |
| 2016 | 17.2 (15.7-18.9) | 12.1 (5.6-25) | 17.5 (15.8-19.6) | 53.8 (53.5-54) | 23.6 (21.9-25.5) | 26.3 (18.3-39.4) | 24.1 (22-26.6) | 67 (66.7-67.3) | 11.5 (10.2-12.9) | 1.4 (0.7-10) | 11.7 (10.4-13.4) | 27.6 (27.4-27.8) | 6.5 (5.6-7.6) | 7.7 (3.0-19.0) | 6.4 (5.5-7.8) | 43.1 (42.9-43.4) | 8 (7-9.2) | 2.7 (0.5-12.3) | 8.1 (7-9.6) | 16.4 (16.3-16.6) |
| 2017 | 17.5 (16-19.1) | 3.2 (1.3-10.9) | 18 (16.4-20.5) | 55.7 (55.4-55.9) | 21.2 (19.7-22.9) | 16 (12-24.5) | 21.9 (19.8-24.8) | 69.8 (69.5-70.2) | 12.8 (11.5-14.2) | 6.5 (2.7-15.7) | 12.9 (11.5-15.1) | 27.4 (27.2-27.6) | 6.5 (5.6-7.5) | 1.9 (0.5-9.5) | 6.6 (5.7-8.6) | 42.9 (42.7-43.1) | 9.1 (8-10.4) | 2.6 (0.2-12) | 9.3 (8.2-11.4) | 16.1 (15.9-16.2) |
