# Supplementary material for: Leading causes of death in Asian Indians in the United States (2005–2017)
Source: PLoS One. 2022 Aug 10;17(8):e0271375. doi: 10.1371/journal.pone.0271375 (PMC9365163; doi:10.1371/journal.pone.0271375)
Supplement: S2 Table — (DOCX) [file pone.0271375.s002.docx]

**Supplemental Table 2. Annual mortality ratio for leading causes of death in Asian Indians and non-Hispanic Whites in the United States by gender, 2005-2017**

| Gender | | | | | | | | | | | | | | | | | | | | |
| --- | --- | --- | --- | --- | --- | --- | --- | --- | --- | --- | --- | --- | --- | --- | --- | --- | --- | --- | --- | --- |
|  | Heart Diseases | | | | Malignant Neoplasms | | | | Cerebrovascular Disease | | | | Diabetes Mellitus | | | | Influenza & Pneumonia | | | |
|  | AI male | AI female | NHW male | NHW female | AI male | AI female | NHW male | NHW female | AI male | AI female | NHW male | NHW female | AI male | AI female | NHW male | NHW female | AI male | AI female | NHW male | NHW female |
| 2005 | 119.6 (110.5-129.4) | 68.3 (61.3-75.8) | 263.5 (262.5-264.5) | 180.1 (179.4-180.9) | 57.5 (51.3-64.4) | 51.2 (45.2-57.8) | 240 (239-240.9) | 171.6 (170.9-172.4) | 15.9 (12.7-19.7) | 17.3 (13.9-21.4) | 45.7 (45.3-46.2) | 49.2 (48.8-49.5) | 18.2 (14.8-22.2) | 14.4 (11.2-18.2) | 36.6 (36.3-37) | 29.4 (29.1-29.7) | 15.5 (12.3-19.3) | 11.1 (8.3-14.5) | 43.9 (43.5-44.3) | 33.8 (33.5-34.1) |
| 2006 | 102.8 (94.8-111.4) | 63.9 (57.2-71.2) | 237.2 (236.2-238.1) | 149.2 (148.6-149.8) | 56.3 (50.4-62.8) | 60.8 (54.1-68.2) | 227.4 (226.5-228.3) | 160.1 (159.5-160.8) | 20.7 (17.1-24.9) | 19.7 (16-24) | 40.4 (40-40.8) | 40.7 (40.4-41) | 20.4 (16.9-24.5) | 14.1 (11.1-17.7) | 33.8 (33.5-34.2) | 25.1 (24.8-25.4) | 16.6 (13.4-20.3) | 13.6 (10.6-17.3) | 39.4 (39-39.8) | 27.1 (26.8-27.3) |
| 2007 | 102.8 (95.3-110.9) | 61.4 (55.4-68) | 228.6 (227.7-229.5) | 143.2 (142.6-143.8) | 53.7 (48.2-59.6) | 54.5 (48.8-60.7) | 224.9 (224-225.8) | 157.4 (156.7-158.1) | 12.5 (10-15.6) | 14.1 (11.3-17.4) | 39.3 (38.9-39.7) | 39.8 (39.4-40.1) | 19 (15.8-22.7) | 14.2 (11.3-17.7) | 33.5 (33.2-33.9) | 24.4 (24.1-24.6) | 14.2 (11.4-17.4) | 9.2 (6.0-12.0) | 37.7 (37.3-38.1) | 26 (25.7-26.2) |
| 2008 | 88.3 (81.6-95.3) | 53.1 (47.9-58.8) | 221.6 (220.7-222.5) | 139.2 (138.6-139.8) | 52.1 (47-57.6) | 51 (45.8-56.8) | 218.8 (217.9-219.7) | 153.2 (152.5-153.9) | 17.9 (15-21.3) | 15.8 (12.9-19.1) | 37.8 (37.4-38.1) | 38.2 (37.9-38.6) | 18.4 (15.5-21.9) | 14.5 (11.7-17.8) | 32.5 (32.2-32.9) | 24 (23.8-24.3) | 13.7 (11.2-16.6) | 12 (9.6-14.9) | 37.7 (37.4-38.1) | 26.6 (26.3-26.9) |
| 2009 | 97.4 (90.7-104.6) | 51.1 (46.1-56.6) | 213.1 (212.3-214) | 130.8 (130.3-131.4) | 58.6 (53.3-64.2) | 49.5 (44.5-54.9) | 215.3 (214.4-216.1) | 150.8 (150.1-151.4) | 13.4 (10.9-16.3) | 16.4 (13.6-19.5) | 36.2 (35.9-36.6) | 36 (35.7-36.3) | 16.9 (14.2-20.1) | 12.4 (10-15.2) | 32.1 (31.7-32.4) | 22.9 (22.7-23.2) | 17.6 (14.8-20.8) | 14.8 (12.2-17.9) | 36.4 (36-36.8) | 25.4 (25.2-25.7) |
| 2010 | 124.9 (115.7-134.8) | 56.2 (51.1-61.8) | 206.3 (205.4-207.1) | 129.5 (129-130.1) | 74.5 (67.8-81.8) | 55.2 (50.1-60.7) | 210.7 (209.9-211.5) | 149.4 (148.7-150) | 23.1 (19.3-27.6) | 16 (13.4-19.1) | 35.3 (35-35.7) | 36.2 (35.9-36.4) | 22.4 (18.8-26.6) | 12.4 (10-15.2) | 31.5 (31.2-31.8) | 22.7 (22.4-22.9) | 22.3 (18.3-26.9) | 12.2 (9.9-14.9) | 35.6 (35.2-35.9) | 24.4 (24.2-24.7) |
| 2011 | 83.3 (77.5-89.5) | 53.3 (48.5-58.5) | 202.4 (201.6-203.2) | 126.9 (126.3-127.4) | 63.4 (58.2-68.9) | 51.4 (46.6-56.6) | 206.2 (205.4-207.1) | 146.4 (145.7-147) | 17.3 (14.7-20.2) | 14.5 (12.1-17.3) | 34.3 (34-34.6) | 35.5 (35.2-35.7) | 19 (16.3-22.1) | 13.1 (10.8-15.8) | 32.9 (32.6-33.3) | 23.6 (23.3-23.8) | 16 (13.5-18.8) | 12.6 (10.4-15.2) | 36.9 (36.6-37.3) | 26 (25.7-26.2) |
| 2012 | 84.3 (78.8-90.2) | 52.7 (48.2-57.6) | 189.6 (188.8-190.4) | 120.8 (120.3-121.4) | 54.2 (49.7-59) | 54.3 (49.7-59.3) | 191.6 (190.9-192.4) | 137.4 (136.8-138) | 15.3 (13.1-18) | 14.9 (12.4-17.6) | 32.1 (31.8-32.5) | 33.6 (33.3-33.9) | 21 (18.2-24.1) | 13.7 (11.4-16.4) | 31.1 (30.8-31.4) | 22.1 (21.9-22.3) | 16.1 (13.8-18.7) | 12.7 (10.5-15.3) | 33.6 (33.3-33.9) | 23.8 (23.6-24) |
| 2013 | 92.3 (86.7-98.1) | 54.6 (50.2-59.4) | 189.2 (188.4-189.9) | 118.8 (118.3-119.3) | 60.1 (55.6-64.9) | 52.5 (48-57.2) | 187.8 (187-188.5) | 135.1 (134.5-135.6) | 15.9 (13.6-18.4) | 14.3 (12.1-16.8) | 32 (31.7-32.3) | 32.7 (32.4-33) | 22.8 (20.1-25.9) | 13.1 (11-15.6) | 31.5 (31.2-31.8) | 22.3 (22.1-22.5) | 19.1 (16.6-21.9) | 14.2 (12-16.7) | 35.8 (35.5-36.2) | 25.6 (25.4-25.9) |
| 2014 | 86.8 (81.6-92.2) | 52.5 (48.3-56.9) | 185.7 (184.9-186.4) | 115.7 (115.2-116.3) | 58.3 (54-62.8) | 52 (47.8-56.5) | 185.5 (184.8-186.2) | 133.4 (132.8-134) | 15.7 (13.5-18.1) | 14.3 (12.2-16.7) | 32.3 (31.9-32.6) | 33.3 (33-33.5) | 18.2 (15.8-20.8) | 12.8 (10.8-15.1) | 32.4 (32-32.7) | 22.4 (22.2-22.6) | 20.2 (17.7-22.9) | 12.5 (10.5-14.8) | 35.1 (34.8-35.5) | 24.9 (24.7-25.2) |
| 2015 | 92 (86.8-97.4) | 55.6 (51.5-60) | 185 (184.2-185.7) | 116.8 (116.3-117.3) | 70.7 (66.2-75.6) | 63.9 (59.3-68.8) | 182.3 (181.5-183) | 131.4 (130.9-132) | 19.3 (16.9-21.8) | 15.2 (13.1-17.6) | 33 (32.7-33.4) | 34.5 (34.3-34.8) | 20.5 (18.1-23.1) | 16.1 (13.9-18.6) | 33.3 (33-33.6) | 23.2 (22.9-23.4) | 19.7 (17.4-22.3) | 16.6 (14.3-19.1) | 35.8 (35.4-36.1) | 25.9 (25.6-26.1) |
| 2016 | 97.2 (92-102.7) | 55 (51-59.3) | 184.5 (183.8-185.3) | 113.6 (113.1-114.1) | 67.3 (62.9-71.9) | 63.6 (59.2-68.3) | 181.7 (181-182.5) | 131.1 (130.5-131.7) | 18.3 (16.1-20.7) | 20.3 (17.9-22.9) | 33.5 (33.1-33.8) | 34.5 (34.2-34.8) | 22.7 (20.2-25.5) | 16 (13.8-18.4) | 33.9 (33.6-34.2) | 23.3 (23.1-23.6) | 20.7 (18.3-23.3) | 15.5 (13.3-17.8) | 34.3 (34-34.7) | 24.3 (24.1-24.5) |
| 2017 | 95.8 (90.7-101) | 55.8 (51.9-60) | 180.1 (179.4-180.9) | 111.7 (111.2-112.2) | 66.1 (61.9-70.6) | 61.3 (57-65.9) | 174.3 (173.6-175) | 126.9 (126.3-127.4) | 18.2 (16-20.5) | 17.7 (15.6-20.1) | 33.2 (32.9-33.5) | 34.5 (34.3-34.8) | 25.7 (23.1-28.5) | 16.3 (14.2-18.7) | 34.4 (34.1-34.7) | 23.9 (23.6-24.1) | 20.5 (18.2-23) | 18.1 (15.8-20.6) | 34.9 (34.6-35.3) | 25.3 (25.1-25.5) |
|  | Heart Diseases | | | | Malignant Neoplasms | | | | Cerebrovascular Disease | | | | Diabetes Mellitus | | | | Influenza & Pneumonia | | | |
|  | AI male | AI female | NHW male | NHW female | AI male | AI female | NHW male | NHW female | AI male | AI female | NHW male | NHW female | AI male | AI female | NHW male | NHW female | AI male | AI female | NHW male | NHW female |
| 2005 | 10 (7.5-13.1) | 6.6 (4.6-9.3) | 41.9 (41.4-42.3) | 44.7 (44.4-45.1) | 25.5 (22.1-29.4) | 10.9 (8.5-13.9) | 80.6 (80-81.1) | 35.4 (35.1-35.7) | 11.6 (9-14.7) | 8 (5.7-11) | 33.9 (33.5-34.2) | 26.1 (25.9-26.4) | 6.7 (4.7-9.4) | 5.2 (3.5-7.7) | 56.7 (56.2-57.2) | 46 (45.7-46.4) | 7 (5-9.7) | 6.1 (4.2-8.7) | 22.3 (22-22.6) | 18.7 (18.5-19) |
| 2006 | 8.7 (6.5-11.5) | 5.9 (4.1-8.4) | 40.1 (39.7-40.5) | 39.2 (38.9-39.5) | 28.6 (25-32.7) | 8.6 (6.4-11.3) | 79.7 (79.1-80.2) | 34.9 (34.5-35.2) | 15.5 (12.7-19) | 7.3 (5-10.2) | 32.4 (32-32.7) | 23.9 (23.6-24.1) | 8.7 (6.4-11.5) | 6.7 (4.7-9.4) | 50.6 (50.1-51) | 39.9 (39.6-40.3) | 8.2 (6.0-11.0) | 9.2 (6.7-12.4) | 20.9 (20.6-21.2) | 16.5 (16.3-16.7) |
| 2007 | 7.3 (5.4-9.7) | 7.2 (5.2-9.6) | 40.6 (40.2-41) | 40 (39.7-40.3) | 26.5 (23.1-30.3) | 11.3 (9-14.1) | 81.2 (80.6-81.7) | 35.7 (35.4-36.1) | 12.2 (9.8-15) | 7 (5.1-9.5) | 32.7 (32.3-33) | 23.6 (23.3-23.8) | 4.9 (3.3-7) | 4.3 (2.9-6.3) | 51.5 (51-51.9) | 40.2 (39.8-40.5) | 8.3 (6.2-11) | 5 (3.4-7.1) | 21.1 (20.8-21.4) | 16.7 (16.5-16.9) |
| 2008 | 7.2 (5.4-9.4) | 7.4 (5.6-9.8) | 42.5 (42.1-42.8) | 41.8 (41.5-42.1) | 24.6 (21.5-28.1) | 11.9 (9.7-14.6) | 80.1 (79.6-80.7) | 35.1 (34.8-35.5) | 11.7 (9.5-14.4) | 6.9 (5.1-9.2) | 32.6 (32.2-32.9) | 23.4 (23.2-23.7) | 6.9 (5.2-9.2) | 5.1 (3.6-7.1) | 54.7 (54.3-55.2) | 43.5 (43.1-43.8) | 6.8 (5.1-9.1) | 4.9 (3.4-7) | 20.3 (20.1-20.6) | 15.4 (15.2-15.6) |
| 2009 | 9 (7.1-11.4) | 6.7 (5-8.9) | 41.9 (41.5-42.3) | 40 (39.7-40.3) | 24.9 (21.9-28.3) | 10.2 (8.3-12.5) | 77.1 (76.6-77.6) | 34.3 (34-34.7) | 11.8 (9.6-14.3) | 7.6 (5.7-9.9) | 31.8 (31.4-32.1) | 22.5 (22.3-22.7) | 6.9 (5.2-9.1) | 5.1 (3.6-7.1) | 52.2 (51.7-52.6) | 41.9 (41.6-42.2) | 7.3 (5.5-9.5) | 6 (4.3-8.2) | 20.1 (19.8-20.4) | 15.3 (15.1-15.5) |
| 2010 | 12.9 (10-16.5) | 8.8 (6.9-11.2) | 42.6 (42.2-43) | 42.2 (41.9-42.6) | 29 (25.5-33) | 9.7 (7.8-12) | 78.4 (77.9-78.9) | 35.8 (35.4-36.1) | 16.4 (13.5-19.8) | 6.9 (5.2-9.1) | 31.7 (31.3-32) | 22.9 (22.6-23.1) | 10.5 (7.8-13.9) | 3.7 (2.5-5.4) | 50.6 (50.2-51) | 41.8 (41.5-42.2) | 11.6 (8.8-15.1) | 5.4 (3.9-7.4) | 20.5 (20.2-20.7) | 15.7 (15.5-15.9) |
| 2011 | 11.7 (9.6-14.1) | 7.6 (5.9-9.7) | 42.9 (42.5-43.2) | 42.6 (42.3-43) | 25.8 (22.9-29) | 9 (7.2-11.1) | 80.3 (79.8-80.8) | 37 (36.7-37.3) | 11.9 (9.8-14.3) | 6.8 (5.1-8.9) | 32 (31.7-32.4) | 23.1 (22.9-23.4) | 7.9 (6.2-10) | 5.4 (4-7.2) | 51 (50.6-51.4) | 42.5 (42.2-42.9) | 6.9 (5.3-8.9) | 6 (4.4-7.9) | 18.9 (18.7-19.2) | 14.6 (14.4-14.8) |
| 2012 | 10.8 (8.9-13) | 7.2 (5.6-9.1) | 41.3 (40.9-41.6) | 40.7 (40.5-41) | 26.4 (23.6-29.5) | 10.9 (9.0-13.0) | 78.9 (78.4-79.5) | 36.7 (36.3-37) | 11 (9.1-13.2) | 6.1 (4.6-8) | 30.5 (30.2-30.8) | 22.2 (21.9-22.4) | 5.7 (4.4-7.4) | 4.5 (3.3-6.1) | 46.9 (46.5-47.3) | 39.9 (39.6-40.2) | 6.1 (4.6-7.8) | 5.4 (4-7.2) | 17.7 (17.5-17.9) | 13.9 (13.7-14) |
| 2013 | 10.8 (9-12.9) | 11.3 (9.3-13.5) | 41.9 (41.5-42.3) | 41.7 (41.4-42) | 25.4 (22.8-28.3) | 8.8 (7.2-10.7) | 79.1 (78.6-79.7) | 37.1 (36.8-37.4) | 12 (10.1-14.2) | 5.7 (4.3-7.4) | 31 (30.7-31.3) | 22.6 (22.3-22.8) | 6.8 (5.4-8.5) | 6 (4.6-7.7) | 47.4 (47-47.7) | 40.8 (40.5-41.1) | 6.4 (5-8.1) | 6.8 (5.2-8.7) | 18 (17.8-18.2) | 14.1 (14-14.3) |
| 2014 | 14.3 (12.3-16.5) | 9.4 (7.7-11.3) | 44.3 (44-44.7) | 44.8 (44.5-45.1) | 25.4 (22.9-28.2) | 10.8 (9.1-12.8) | 81.2 (80.7-81.7) | 38 (37.7-38.4) | 11.3 (9.5-13.4) | 7 (5.5-8.8) | 31.3 (31-31.7) | 22.7 (22.5-23) | 7.2 (5.8-8.9) | 4.3 (3.2-5.7) | 45.8 (45.4-46.2) | 39.3 (39-39.6) | 8 (6.5-9.8) | 6.5 (5.1-8.2) | 18.4 (18.1-18.6) | 14.2 (14-14.4) |
| 2015 | 16.7 (14.6-19.1) | 12.7 (10.8-14.9) | 49.4 (49-49.8) | 51 (50.6-51.3) | 28.9 (26.3-31.8) | 14.3 (12.3-16.6) | 85.6 (85.1-86.2) | 40.1 (39.7-40.4) | 14 (12-16.1) | 7.9 (6.4-9.6) | 31.7 (31.4-32.1) | 23.5 (23.2-23.7) | 6.1 (4.8-7.6) | 5.1 (3.9-6.5) | 46.6 (46.3-47) | 41.1 (40.8-41.4) | 8 (6.5-9.7) | 7.8 (6.3-9.7) | 18.9 (18.7-19.1) | 14.5 (14.3-14.7) |
| 2016 | 18.3 (16.1-20.7) | 16 (13.9-18.3) | 51.9 (51.5-52.3) | 54.4 (54.1-54.8) | 31.9 (29.1-34.8) | 14.8 (12.8-17) | 92.7 (92.2-93.2) | 42.3 (42-42.7) | 15.1 (13.1-17.3) | 7.5 (6.1-9.3) | 31.9 (31.6-32.3) | 23.6 (23.3-23.8) | 8 (6.6-9.6) | 5 (3.9-6.4) | 46.6 (46.3-47) | 40.6 (40.3-40.9) | 8.9 (7.4-10.6) | 7.1 (5.7-8.8) | 19 (18.8-19.2) | 14.6 (14.5-14.8) |
| 2017 | 19.1 (16.9-21.5) | 15.8 (13.7-18) | 53.5 (53.1-53.9) | 56.6 (56.2-56.9) | 31.3 (28.6-34.2) | 10.4 (8.8-12.2) | 96.5 (95.9-97) | 44.2 (43.8-44.5) | 15.7 (13.7-17.9) | 9.6 (8-11.6) | 31.5 (31.2-31.8) | 23.6 (23.3-23.8) | 7.5 (6.2-9.1) | 5.4 (4.3-6.8) | 45.8 (45.4-46.1) | 40.9 (40.6-41.2) | 10.2 (8.6-12.1) | 8 (6.5-9.7) | 18.5 (18.3-18.7) | 14.3 (14.1-14.5) |
