# Supplementary material for: Leading causes of death in Asian Indians in the United States (2005–2017)
Source: PLoS One. 2022 Aug 10;17(8):e0271375. doi: 10.1371/journal.pone.0271375 (PMC9365163; doi:10.1371/journal.pone.0271375)
Supplement: Attachment — Submitted filename: Response to Reviwers.docx [file pone.0271375.s003.docx]

PONE-D-21-08873 Manuscript Revisions 1

Dear Dr. Peter Tugwell and Dr. David I. Tovey,

Thank you for considering our manuscript, PONE-D-21-08873 “Leading Causes of Death in Asian Indians in the United States (2005 - 2017)” for publication. We appreciate the opportunity to revise our manuscript and the helpful comments from the reviewers that have allowed for our manuscript to be clearer and more informative. The corrections made are as follows:

**Comments from Reviewer 1:**

1. Have the authors made all data underlying the findings in their manuscript fully available? No.

**Response:** After looking over the PLOS data policy and our data, since our data is de-identified, we are going to submit it along with the manuscript. Additional information on this topic can be found in the methods section.

2. It was a little difficult to understand the age of mortality item in the earlier section of the paper though it is explained in the discussion. Maybe where it is first introduced, the authors might refer the reader to the later discussion or give a brief explanation?

**Response:** We removed that specific term from the earlier part of the paper in order to explain more clearly in the results section that the average age of mortality was over double for foreign-born Asian Indians as compared to US-born Asian Indians since only a small percent of them were over 65 years.

3. Additionally, now that the 2020 Census data are coming out, it could be good to note that and state additional examination of mortality for this key population should continue for the data to come using the 2020 population denominators etc.

**Response:** Thank you for pointing this out, we have added a statement about it in the conclusions section of our paper mentioning how further research into mortality rates using that data can be done.

**Comments from Reviewer 2:**

1. The authors should provide a citation about the drivers of immigration for Asian Indians, both in the 1960s and more recently. The claims are made without references.

**Response:** We have included two sources to support our statements regarding immigration of Asian Indians in the introduction section. Both references can be found in the appropriate section as reference 36 and 37.

2. The authors assert that blending of Asian Indian and American cultures may impact risks, but don't expand upon why specifically for this population group. In addition, they also state that this consideration supports "place of birth" as an important health determinant, which implies the presumption that this is only or primarily true for immigrants.

**Response:** Clarification has been provided on the contextualization of the “place of birth” assertion made in the text, expanding on the importance of taking this determinant into account beyond just immigrants. Further explanation has also been provided on the first assertion throughout and in the discussion section.

3. Outside of being "in accordance with survey data", the rationale for special age brackets is unclear. Please provide more context.

**Response:** More context has been provided in the methods section, namely that we created age brackets for the census data to match up to the survey data. Thank you for pointing this out.

4. The US-born Asian Indian average age of death seems shocking low (35.1) and it is unclear how Figure 4 makes this clear (the title of the figure reflects age distributions by sex for selected endpoints). I would urge the authors to make sure that value is accurate. If so, this requires some attention in the Discussion.

**Response:** This value is indeed correct; however, we cited the wrong figure. Figure 3 is the one that helps give that value (the title is “Age distribution for leading causes of death in foreign-born and US-born Asian Indians, 2005-2017”). Furthermore, this result has been elaborated on in the results as well as in the discussion sections, offering a potential reason as “The significantly lower average age of death for US-born Asian Indians arises from the younger age demographics of this group, explained by historical and migratory trends”.

5. *Building off the prior comment, I find the Discussion to be primarily a repeat of the results. Although it was not measured in this study, I would like to see the results placed in more nuanced context of the extant literature. Especially with respect to modifiable risk factors that have socio-cultural underpinnings, these need to be described more fully (currently there are generic statements about diet, physical activity, genetics, and response to medication). If the reader is to be a health professional, what do these results mean for intervention and prevention?

**Response:** These have been further elaborated on in the discussion section. It has been acknowledged that the decrease in heart disease mortality for US-born Asian Indians compared to foreign-born Asian Indians implies some influence of cultural and socio-economic factors. Specifics have been given, such as barrier to access to healthcare particularly in foreign-born Asian Indians, and interventions targeting specific sub-group issues and improving surveillance to better represent the mentioned ethnic groups are suggested.

6. Similarly, more detail about differential distributions of cancer among Asian Indians would be beneficial, as this study treats cancer death as a singular outcome.

**Response:** More details are given regarding differences in cancer mortality rates by nativity, and figure 3 is cited as showing a large heterogeneity in age distributions, leading to different types of cancer having differing mortality rates among the groups.

7. The idea that this study is in contrast with a "healthy immigrant" effect is compelling, and I wish more attention was provided to elucidate this; currently, the authors make general comments about generational and cultural differences related to behavior, along with health care access differences. I think an opportunity is lost for this paper to provide concrete directions for clinical and public health practice, as opposed to a reiteration of the findings in the final sections of the manuscript.

**Response:** Thank you for this comment. Similarly to comment number five, we have gone into further detail about interventions and what can be done in the discussion and conclusion sections given the contrast to the “healthy immigrant” effect and how specific barriers that might have influenced this result need to be properly addressed.

We thank the reviewers once again for the comments, they were helpful in focusing our paper on specific issues and being able to elaborate on those further. We look forward to hearing from you and we are prepared to make further revisions if necessary.

Sincerely,

Aditya V. Simha

Stanford Center for Asian Health Research and Education (CARE)

Stanford University School of Medicine

Department of Neurobiology, Physiology and Behavior, University of California, Davis

[asimha@stanford.edu](mailto:asimha@stanford.edu) / [avsimha@ucdavis.edu](mailto:avsimha@ucdavis.edu)

**Additional Manuscript Authors:**

Claudia Fernandez Perez

Stanford Center for Asian Health Research and Education (CARE)

Stanford University School of Medicine

Kevin Xi

Stanford Center for Asian Health Research and Education (CARE)

Stanford University School of Medicine

Malathi Srinivasan, MD FACP

Clinical Professor of Medicine

Division of Primary Care and Population Health

Stanford University School of Medicine

[malathis@stanford.edu](mailto:malathis@stanford.edu)

Nilay S. Shah, MD, MPH

Instructor of Medicine (Cardiology) and Preventive Medicine

Northwestern University Feinberg School of Medicine

[nilay.shah@northwestern.edu](mailto:nilay.shah@northwestern.edu)

Latha Palaniappan, MD, MS, FAHA, FACC, FACP

Professor of Medicine

Stanford University School of Medicine

[lathap@stanford.edu](mailto:lathap@stanford.edu)
