# Supplementary material for: Leading causes of death in Asian Indians in the United States (2005–2017)
Source: PLoS One. 2022 Aug 10;17(8):e0271375. doi: 10.1371/journal.pone.0271375 (PMC9365163; doi:10.1371/journal.pone.0271375)
Supplement: Attachment — Submitted filename: Response to Revisions 3.docx [file pone.0271375.s004.docx]

PONE-D-21-08873 Manuscript Revisions 3

Dear Dr. Peter Tugwell and Dr. David I. Tovey,

Thank you for considering our manuscript, PONE-D-21-08873 “Leading Causes of Death in Asian Indians in the United States (2005 - 2017)” for publication. We appreciate the opportunity to revise our manuscript and the helpful comments from the reviewers that have allowed for our manuscript to be clearer and more informative. The last update according to the suggestion made is as follows:

1. For the most part, the concerns I articulated in my initial review have been addressed. However, in the Discussion, I would have liked to see more specificity in the recommendations for public health practice. For instance, there is a wealth of literature looking at specific dietary patterns among Asian Indians that could have been emphasized, or social/cultural barriers to cancer screening. Currently, the implications for disease prevention and treatment still remain in the realm of generic recommendations which could be applied to any population group.

   I defer to the editors to determine if and how this section could be more concrete and targeted, given the population-specificity of this work.

   I look forward to seeing this manuscript as a publication in the base of peer-reviewed literature in the near future.

**Response:** Thank for suggesting this review to strengthen the discussion section and recommendations to healthcare practitioners. Additional information has been incorporated to reflect the notable cancer disparities among Asian Indians, as well as the sociocultural barriers that they face. It’s also been pointed out that there is crucial need for further research into how this translates into differences in cancer related mortality between foreign-born and US-born Asian Indians. We had also incorporated information about specific diet related and other lifestyle factors that can be at play in the broad disparities that we see among this population. We further note how these findings can guide further research and practitioners in developing culturally sensitive practices that reduce these disparities.

We thank the reviewers once again for the comments, they were very helpful in focusing and strengthening our manuscript and furthering the population-specific impact it will have. We look forward to hearing from you and we are prepared to make further revisions if necessary.

Sincerely,

Claudia Fernandez Perez

Stanford Center for Asian Health Research and Education (CARE)

Stanford University School of Medicine

Johns Hopkins Bloomberg School of Public Health

[cxf507@miami.edu](mailto:cxf507@miami.edu) / [cferna36@jh.edu](mailto:cferna36@jh.edu)

**Additional Manuscript Authors:**

Aditya V. Simha

Stanford Center for Asian Health Research and Education (CARE)

Stanford University School of Medicine

Department of Neurobiology, Physiology and Behavior, University of California, Davis

[asimha@stanford.edu](mailto:asimha@stanford.edu) / [avsimha@ucdavis.edu](mailto:avsimha@ucdavis.edu)

Kevin Xi

Stanford Center for Asian Health Research and Education (CARE)

Stanford University School of Medicine

Malathi Srinivasan, MD FACP

Clinical Professor of Medicine

Division of Primary Care and Population Health

Stanford University School of Medicine

[malathis@stanford.edu](mailto:malathis@stanford.edu)

Nilay S. Shah, MD, MPH

Instructor of Medicine (Cardiology) and Preventive Medicine

Northwestern University Feinberg School of Medicine

[nilay.shah@northwestern.edu](mailto:nilay.shah@northwestern.edu)

Latha Palaniappan, MD, MS, FAHA, FACC, FACP

Professor of Medicine

Stanford University School of Medicine

[lathap@stanford.edu](mailto:lathap@stanford.edu)
